# Epidemiology and Cost of Nosocomial Gastroenteritis, Avon, England, 2002–2003

**DOI:** 10.3201/eid1010.030941

**Published:** 2004-10

**Authors:** Ben A. Lopman, Mark H. Reacher, Ian B. Vipond, Dawn Hill, Christine Perry, Tracey Halladay, David W. Brown, W. John Edmunds, Joyshri Sarangi

**Affiliations:** * Health Protection Agency, London, UK;; † Health Protection Agency, Bristol, UK;; ‡ United Bristol Healthcare Trust, Bristol, UK;; § North Bristol NHS Trust, Bristol, UK;; ¶ Royal United Hospital Bath NHS Trust, Bath, UK

**Keywords:** gastroenteritis, nosocomial, outbreak, norovirus, norwalk, healthcare-acquired infection, infection control, economic, health economics, research

## Abstract

Healthcare-associated outbreaks of gastroenteritis are an increasingly recognized problem, but detailed knowledge of the epidemiology of these events is lacking. We actively monitored three hospital systems in England for outbreaks of gastroenteritis in 2002 to 2003. A total of 2,154 patients (2.21 cases/1,000-hospital-days) and 1,360 healthcare staff (0.47 cases/1,000-hospital-days) were affected in 227 unit outbreaks (1.33 outbreaks/unit-year). Norovirus, detected in 63% of outbreaks, was the predominant etiologic agent. Restricting new admissions to affected units resulted in 5,443 lost bed-days. The cost of bed-days lost plus staff absence was calculated to be £635,000 (U.S.$ 1.01 million) per 1,000 beds. By our extrapolation, gastroenteritis outbreaks likely cost the English National Health Service £115 million (U.S.$ 184 million) in 2002 to 2003. Outbreaks were contained faster (7.9 vs. 15.4 days, p = 0.0023) when units were rapidly closed to new admissions (<4 days). Implementing control measures rapidly may be effective in controlling outbreaks.

## Abstract 2

Implementing control measures rapidly may be effective in controlling gastroenteritis outbreaks.

Nosocomial gastroenteritis outbreaks, particularly those caused by noroviruses, have become increasingly important in Europe (*1*) and North America and have attracted media interest (*2**–**4*). However, unlike bloodstream, surgical-site, respiratory, skin, and urinary tract infections, tools for detecting and measuring hospital-associated gastroenteritis outbreaks have not been well developed, thus precluding accurate measurement of incidence and cost of these infections (*5**–**8*).

In England and Wales, the Health Protection Agency Communicable Disease Surveillance Centre has operated a passive surveillance system for gastroenteritis outbreaks. From 1992 to 2000, information was collected on >5,000 outbreaks, 27% of which occurred in hospitals and 28% in residential facilities, primarily nursing homes (*1**,**3*). Of these outbreaks, >50% were caused by norovirus and 25% were presumed viral on the basis of clinical signs and symptoms and outbreak characteristics, including high frequency of vomiting, short duration of illness, and short incubation period (*1*). Particular patterns of transmission have been observed in the outbreaks in healthcare facilities, in which economic effects are likely to be considerable (*3*).

Because noroviruses are the most common cause of gastroenteritis in the community (*9*), keeping the virus from being introduced into healthcare settings is difficult, particularly in winter months. For this reason, control measures focus on minimizing the spread of virus within and between hospital units (*10*). Closing a unit to new patient admissions, excluding affected staff from work for 48 hours postrecovery, and rigorous disinfection are the key features of current control guidelines.

Studies have reported that annually 20%–25% of the population has gastroenteritis (*11*); however, these surveys excluded persons in healthcare facilities (*9*). Although many hospital outbreaks of gastroenteritis have been described (*12**–**14*), information from systematic, population-based surveillance of gastroenteritis in healthcare settings is lacking (*10*).

We performed active surveillance of hospital outbreaks of gastroenteritis to determine incidence, microbiologic cause, economic cost, and effectiveness of control measures in the county of Avon, England, an area likely to be broadly representative of England as a whole.

## Methods

### Surveillance System

#### Clinical Definitions

**Figure 1:**
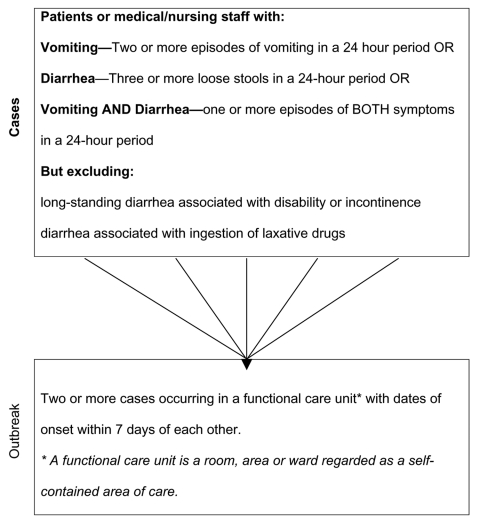
Definition of an outbreak of gastroenteritis in healthcare settings.

Since this surveillance system is designed for detecting outbreaks of gastroenteritis, a two-tiered definition (of cases and outbreaks) was required (Figure 1). These definitions, which draw on Kaplan's criteria of an outbreak of viral gastroenteritis (*15*), were developed in consultation with public health professionals at all levels of infection control. Ethical approval for this work was obtained from the South West Multi-centre Research Ethics Committee.

#### Study Population

Gastroenteritis, particularly of viral etiology, is inconsistently reported (*16*). The county of Avon, England, was selected to focus efforts on collecting complete, high-quality data. The all-cause, age-standardized death rate, and deprivation measures and age distribution indicate that the population of Avon is very similar to that of the whole of England and Wales (http://www.avon.nhs.uk).

Three National Health Service administrations (known as NHS Trusts), comprising four major acute hospitals (similar to secondary/tertiary hospitals in the United States) and 11 smaller community hospitals (similar to primary-level hospitals in the United States) that operate in the sentinel area, were monitored under the surveillance network. Combined, these hospitals have 2,900 inpatient beds, which, on average, maintain 95.6% occupancy of their acute-care beds. In total, 171 "functional care units" were monitored; these units were defined as a room, area or ward regarded as a self-contained area that monitored 171 inpatients. The median number of beds on an inpatient unit was 20 (range 1–38), which reflects the large size of units in NHS hospitals compared to those in many other European or North American designs.

Nursing, medical, and other staff members were included in the population at risk. Time-at-risk for staff members was collected from whole-time equivalent staffing levels supplied by human resources departments. Time-at-risk for patients was calculated by using bed occupancy data from the administration system.

#### Surveillance and Outbreak Investigation

Each NHS Trust has an infection control team that includes a medically trained microbiologist, a senior infection control nurse, and a team of dedicated infection control nurses. A total of 11 infection control nurses worked at the three trusts. Infection control nurses were responsible for monitoring the populations in their hospitals. Infection control nurses became aware of outbreaks during ward rounds or were alerted to incidents by nurses working on wards. When an event occurred that met the definition of an outbreak (Figure 1), institutions were requested to contact the study coordinator at the Health Protection Agency in Colindale, London. The study coordinator was responsible for ensuring completeness of reports, overseeing data entry, and performing analyses. The study coordinator also solicited monthly null reports in months that no outbreaks were reported in order to confirm that no outbreaks occurred.

#### Sampling and Diagnostics

Staff members who managed outbreaks were asked to take specimens from the first 10 patients in an outbreak for virologic analysis and from the first 3 patients for bacterial analysis. Such a large number was suggested because of the low sensitivity of viral diagnostics (*17*). Fecal specimens were preferred, but vomit samples were also accepted for virologic testing. Explicit instructions, based on the Health Protection Agency standard operating procedure (*18**–**19*), about taking and sending the samples, were provided. Specimens were tested for viral pathogens at the regional public health laboratory. Specimens were first screened with an in-house enzyme-linked immunosorbent assay (ELISA), followed by reverse transcription-polymerase chain reaction (RT-PCR) for detection of norovirus (*20**,**21*).

#### Outbreak Data

Case forms and an outbreak summary form were completed by infection control nurses as an outbreak progressed. Forms were returned by mail shortly after an outbreak ended. The duration of an outbreak was calculated as the number of days from the onset of the first case to the onset of the last case.

### Statistical Analysis

Data were entered and stored in an Access (Microsoft, Redmond, WA) database. Analyses were performed on Microsoft Excel and Stata 8.0 (*22*). The t-test was used to compare means; the χ^2^ test was used to compare proportions. Continuous data were analyzed with linear regression. Spearman rank test was used to assess correlation of seasonal patterns.

### Economic Analysis

The National Health Service of England is a socialized healthcare system. Funding originates from taxpayer money and is distributed by the Department of Health. Resources are allocated to Primary Care Trusts, which commission hospital services from NHS Hospital Trusts (*23*). Allocations are based on the age distribution of the population served by the hospitals, with adjustments made for maternal needs, mental health, and ambulatory needs of the population (*24*). Thus, funding is not directly based on the services provided. If healthcare provision is disrupted by an avoidable event, such as hospital-acquired infection, the allocated resources are not used optimally. In other words, opportunity costs (the difference between actual performance of an investment and the optimum expected outcome) are incurred.

We analyzed the opportunity costs of nosocomial gastroenteritis outbreaks to the healthcare service and lost productivity of patients (and the families of pediatric patients). Bed-day loss from new admission restriction for affected units and staff absence from illness were estimated as the two main costs related to gastroenteritis outbreaks in hospitals. Other possible economic effects may include cancelled operations, overuse of beds caused by delayed discharge, additional cleaning procedures, and increased drug prescribing. However, these costs are probably limited, since the illness is relatively short-lived, cleaning is a relatively minor expense, and no treatment is available for viral gastroenteritis except rehydration. We also estimated the societal cost of lost productivity from missed days of work. Intangible costs, such as pain and psychological distress from delayed or cancelled operations and admissions, which are difficult to quantify (*25*), were not calculated.

Figures from the Unit Costs of Health and Social Care 2002 report were used to estimate the economic loss from empty beds and staff absence (*26*). Average wage estimates for England were obtained from the Office of National Statistics (*27*). All costs are in Great British pounds (2002) and converted to U.S. dollars at the rate of £1: $1.6, based on the 5-year average 1999–2003 (http://www.forexdirectory.net/home.html).

For economic estimates, the following assumptions were made. Staff members were, on average, grade E nurses, the mid-range of NHS nursing staff. This figure is probably an underestimate of cost since medical staff, who have higher wages, were also affected in outbreaks. A lost bed-day is a real economic loss in terms of opportunity cost. Since these trusts operate at >95% occupancy of inpatient beds, the result is bed-days lost because the bed would likely have been used. These expenditures cannot be reallocated since infection control guidelines stipulate that staff members from affected units are not to work on unaffected units, and patients from affected units are not to be transferred to unaffected units (*10*).

Patients of working age (18–64 for men and 18–59 for women) and one family member of each pediatric patient (<18 years) were assumed to be economically active. We assumed that 5 of 7 days of work were missed for each day of illness in these categories. This figure is an overestimate since many days of hospital-acquired gastroenteritis illness would have been spent in the hospital whether the person acquired gastroenteritis or not. Individual length-of-stay data were not available. Hospital staff absence was considered a cost to the healthcare sector, rather than society.

## Results

### Outbreaks, Cases, and Incidence

**Figure 2:**
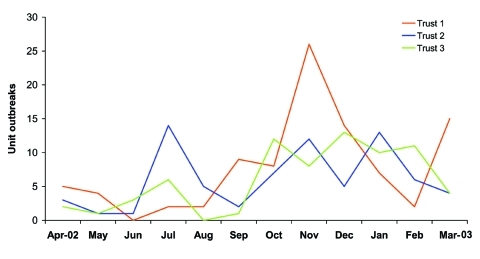
Monthly outbreaks of gastroenteritis in hospitals: Avon, England, April 2002–March 2003 (n = 227).

**Figure 3:**
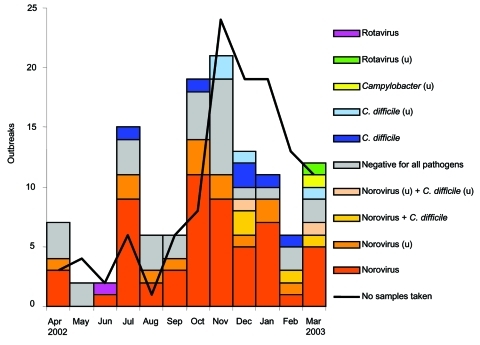
Monthly distribution of outbreaks with diagnostic results (n = 122). Negative outbreaks followed a similar seasonal pattern to norovirus outbreaks. u, unconfirmed (only one positive specimen).

In the 171 inpatient units followed, a total of 227 outbreaks occurred; the outbreak incidence was 1.33 outbreaks per unit-year of risk (95% confidence interval [CI] 1.16–1.51) (Figure 2 and Figure 3). All enrolled hospital trusts were affected by outbreaks. Hospital outbreaks peaked in November, with 46 affected units. A smaller peak occurred July, with 22 outbreaks.

Within the 227 outbreaks, 2,154 hospital patients and 1,360 hospital staff met the case definition. The incidence among patients was 2.21 cases per 1,000 hospital-days at risk (95% CI 2.16–2.25) and among staff was 0.47 cases per 1,000 hospital-days at risk (95% CI 0.45–0.50). Units with outbreaks were significantly larger than those that did not have an outbreak in the study period (21.4 vs. 12.6, p value < 0.0001, t-test).

### Diagnostic Results

Specimens were taken for diagnostic analyses in 122 (51%) of the 227 hospital unit outbreaks (Table 1). Norovirus was the confirmed etiologic agent in 61 outbreaks (50%) and was detected in a single specimen in 16 outbreaks (13%). The second most prevalent organism was *Clostridium difficile*, which was confirmed in nine outbreaks (7%) and detected in a single sample in eight outbreaks (6.5%). Six outbreaks (4.9%) occurred in which both norovirus and *C. difficile* were detected. Rotavirus and *Campylobacter* outbreaks were also detected. Outbreaks from which all specimens were negative for rotavirus and *Campylobacter* (n = 31, 25%) had a similar seasonal pattern to norovirus outbreaks (p = 0.13, Spearman rank test) (Figure 3). The monthly distribution of outbreaks in which no specimens were taken correlated with the monthly distribution of norovirus-confirmed outbreaks.

**Table 1:** Causative organism in hospital gastroenteritis outbreaks, Avon, England, April 2002–March 2003

Organisms	Outbreaks		
	N	%	Combined %
Norovirus^a^	57	46.7	63.1
Norovirus^b^	14	11.5	
Norovirus^a^ + *Clostridium difficile^c^*	4	3.3	13.9
Norovirus^b^ + *C. difficile^a^*	2	1.6	
*C. difficile^b^*	6	4.9	
*C. difficile^a^*	5	4.1	
Rotavirus^a^	1	0.8	
*Campylobacter* ^b^	1	0.8	
Rotavirus^b^	1	0.8	
Negative specimens	31	25.4	
Total with sufficient samples^a^	122		

^a^Confirmed outbreaks (two or more positive specimens). Two or more samples were taken in 122 (54%) of 227 outbreaks. ^b^Unconfirmed outbreaks (single positive specimen)

#### Attack Rates within Outbreaks

In hospital outbreaks, attack rates among staff members (staff affected/staff working on unit: 19.6%, 95% CI 16.6%–22.7%) were significantly lower than those of patients (patients affected/unit beds: 46.8%, 95% CI 40.9%–52.8%) (p < 0.001, t-test). In outbreaks in which norovirus was the confirmed etiologic agent, attack rates were somewhat higher than all outbreaks at 24.5% for staff (95% CI 17.8%–31.2%) and 53.2% for patients (95% CI 41.5%–65.0%), although not significantly so. Attack rates among staff were not higher in the first outbreak (20.6%; 95% CI 16.4%–25.0%) compared to subsequent outbreaks that occurred in the same unit (21.5%; 95% CI 17.2–25.9) (p = 0.8; t-test).

#### Closing Units to New Admissions and Bed-Day Loss

One hundred and fifty-eight (69.6%) of the 227 hospital unit outbreaks resulted in the affected unit's being closed to new admissions (Table 2). Outbreaks in which norovirus was detected did not result in unit closure to new admissions more frequently than outbreaks in which diagnostic results were negative (71.3% compared to 70.6%, respectively) (p = 0.9, χ^2^ test).

**Table 2:** Characteristics of hospital outbreaks of infectious intestinal disease, Avon, England, April 2002–March 2003^a^

Characteristic	Total
Total inpatient wards followed-up	171
Inpatient unit outbreaks^b^	227
Incidence (outbreaks per unit year) (95% CI)	1.33 (1.16–1.51)
Duration of outbreak	
Mean days per outbreak (95% CI)	9.21 (6.54–11.88)
Unit Closure	
Number of unit closures to new admissions (% of all outbreaks)	158 (69.6)
Total number of days of closure to new admissions	1,527
Mean number of days closed per closure to new admissions (95% CI)	9.65 (8.50–10.81)
Mean number of bed days lost^c^ per day of closure to new admissions (95% CI)	3.57 (1.86–5.23)
Total bed days lost^d^ (95% CI)	5,443 (2,838–7,968)

^a^CI, confidence interval. ^b^Eleven outbreaks occurred in outpatient units and affected staff members only. ^c^Beds that remained empty because the unit was closed to new admissions. This number does not include beds blocked because patient could not be discharged because of the outbreak. ^d^Days of unit closure to new admissions x bed-days lost per day of unit closure.

Units were closed for a mean of 9.65 (95% CI 8.5–10.8) days, but in the most extreme example, a unit was closed to new admissions for 48 days because of a single outbreak. On average, 3.57 (95% CI 1.86–5.2) bed-days were lost for every day of unit closure to new admissions, which resulted in an estimated 5,443 bed-days lost from gastroenteritis outbreaks.

### Economic Loss

Unit closures to new admissions were distributed among unit type specialties (Table 3). The cost of empty beds to the three hospitals was £1.49 million (U.S.$ 2.24 million) or approximately £480,000 (U.S.$ 768,000) per 1,000 beds.

**Table 3:** Hospital unit closure to new admissions and economic loss from empty beds, Avon, England, April 2002–March 2003

Unit	Outbreaks resulting in unit closure to new admissions	Total days of closure	Cost per inpatient bed-day^23^	Total cost (GBP)^a^
Admissions	5	47	273	45,807
Cardiology	11	119	460	195,422
Ear Nose and Throat	1	12	273	11,695
Endocrinology/Diabetes	3	58	273	56,527
Geriatric	26	258	145	133,554
Gynocology	0	2	273	1,949
Intensive Care	1	8	273	7,797
Medical	49	496	273	483,407
Mental Health	5	61	177	38,545
Neurology/Neurosurgery	5	63	272	61,176
Obstetrics/Maternity	1	2	273	1,949
Oncology/Radiology	1	5	354	6,319
Orthopaedic/Ortho Trauma	17	109	273	106,232
Pediatric	4	38	398	53,993
Rehabilitation	2	22	192	15,080
Renal	1	5	273	4,873
Respiratory	8	78	273	76,020
Rheumatology	1	3	241	2,581
Surgery	17	141	368	185,240
Total	158	1527		£1,488,165 (US$ 2,381,064)

^a^Total days of closure to new admissions x mean days of bed loss per day of closure (3.57 days of bed-loss per day of closure) x cost per inpatient bed-day. GBP, Great British pounds.

Costs associated with staff absence were calculated as shown in Table 4. A total of 1,360 infections were in staff members; mean duration of illness was 2.4 days. Hospital staff members were advised not to work for 2 days after recovering from gastrointestinal illness (*10*). If the staff members work 5 days a week, an estimated 3.14 days of work were missed because of illness [(2.4 days ill + 2 days absence postrecovery) x (5 working days/7 days)]. The cost of one day absence was £113 (U.S.$ 181); therefore, outbreaks cost £482,000 (U.S.$ 771,000) or £156,000 (U.S.$ 249,000) per 1,000 beds. Total cost of bed-day loss and staff absence was £1.97 million (U.S.$ 3.15 million), or £635,000 million (U.S.$ 1.01 million) per 1,000 beds.

**Table 4:** Costs associated with staff absence from nosocomial outbreaks of infectious intestinal disease, Avon, England, April 2002–March 2003

Row	Item	Figure
A	Number of staff cases	1,360
B	Mean duration of illness	2.4
C	Recommended days staff should remain absent following recovery	2
D	Weekly proportion of days worked	5:7
E	Daily cost of NHS nurse^a^	£113
	Total cost of staff absence^b^	£482,944 (US$ 794,110)

^a^National average cost based on mid-point of grade E nurse. ^b^A x (B + C) x D x E.

A total of 971 days of illness occurred among working age men (433 days), working age women (241 days), and children <18 years of age (297 days). Therefore, 139 (971/5 x 5/7) 5-day work weeks were potentially lost. At £476 (U.S.$ 761) per week, total productivity loss is estimated to be £66,000 (U.S.$ 106,000) or £22,700 (U.S.$ 36,400) per 1,000 beds.

#### Restricting New Admissions

Information about unit closure was available for 52 (85%) of the 61 norovirus-confirmed outbreaks. Forty-nine (94%) of these outbreaks resulted in the unit's being closed to new admissions, but only 7 (13.7%) were closed within 3 days of the date of onset of the primary case. Outbreaks in which the affected unit was closed within the first 3 days were contained in a mean 7.9 days (95 % CI 4.3–11.5); outbreaks in units that were not closed or were closed >3 days after the first case lasted for a mean of 15.4 days (95% CI: 13.6–17.3) (p value = 0.002, t-test). Although not reaching levels of statistical significance, the attack rates for patients (0.52 compared to 0.68, p = 0.38), staff members (0.14 compared to 0.27, p = 0.21), and all cases (16.3 compared to 23.7, p = 0.065) all increased if the unit was not shut within 3 days. Units closed within 3 days of outbreak were not different from other units in terms of size, according to linear regression models. Unit size and specialty did not affect the estimated cost of closing the unit to new admissions.

## Discussion

In our study, the first published systematic assessment of healthcare-associated gastroenteritis outbreaks (primarily caused by noroviruses), we have demonstrated the cost of such outbreaks. On average, each hospital unit (or ward) had 1.33 outbreaks in the 1-year follow-up period. To control the spread of disease as recommended in national guidelines (*10*), 158 of the 227 outbreaks resulted in closing the unit to new admissions. This closure resulted in 5,443 lost bed-days, »0.5% of all available acute bed-days. This bed loss, combined with staff absence, cost an estimated £635,000 (U.S.$ 1.01 million) per 1,000 beds. The measures taken to control the outbreak are costly, but these data indicate that they may be effective in controlling the duration of an outbreak. Units closed within the first 3 days of an outbreak are contained faster than those not closed or closed after day 4 (7.9 vs. 15.4 days; p = 0.002).

The incidence rates in hospital patients and hospital staff were determined to be 2.21 and 0.47 cases per 1,000 hospital-days of risk, respectively. In other words, a patient who spent a year in the hospital would have an 80% chance of having a case of gastroenteritis during an outbreak. This estimate translates to a 1.5% chance for the average inpatient length of stay (≈ 7 days). Full-time hospital staff members had a 17% chance of being affected during the year of follow-up. Norovirus was the predominant etiologic agent detected in 63% of hospital unit outbreaks.

The strength of this study and the high quality of data collected were due to the active and systematic approach. The definitions were designed to ascertain outbreaks by using a clear designation of the spatial boundaries. Thus, if infection spread from one unit to another, the events were counted as two separate outbreaks. However, the role of sporadic gastroenteritis in healthcare settings was not assessed in this study. The study team applied standard case and outbreak definitions. Null reporting was used on a monthly basis to confirm that an outbreak had not occurred when none was reported. The full range of modern diagnostics for viral gastroenteritis, including ELISA and RT-PCR assays, was used. However, even using these tests, viral pathogens are not always identified (*17*). The seasonal pattern of outbreaks in which all specimens were negative in this study suggests that many may also have been caused by noroviruses.

In 2002, norovirus epidemics occurred in the United States, England, Wales, and the rest of Europe (*28**,**29*), raising the question: Are the figures reported here representative or the product of an anomalous year? For this reason, this surveillance will continue in forthcoming years to determine whether the cost of the 2002–2003 season is characteristic or not.

Noroviruses are the predominant agent for outbreaks of gastroenteritis in healthcare settings, a finding that is consistent with previous studies from the United States (*4*), Sweden (*30*), and historical surveillance data from England and Wales (*1**,**3*). However, our study extends these etiologic studies by determining the economic cost of gastroenteritis outbreaks and incidence rates in a defined population. The rates of infection were highest in children and the elderly (*31*). We could not assess whether length of stay was directly affected by hospital-acquired gastroenteritis or whether death rates increased, since these data were not available.

At U.S.$ 1 million per 1,000 beds, the direct costs to the health sector are substantial and outweigh the indirect cost of lost productivity (overestimated to be U.S.$ 36,400 per 1,000 beds.) This proportional cost to health service is largely because of the age distribution of hospital populations that were affected: <20% of patients were pediatric patients or economically active. Other healthcare sector costs, such as cancelled operations, bed blocking from delayed discharge, additional cleaning procedures, and increased drug prescribing, could be estimated in future studies. However, these costs are probably limited since the illness is relatively short-lived, cleaning is a relatively minor expense, and viral gastroenteritis has no treatment except rehydration. For these reasons, the indirect cost of nosocomial gastroenteritis outbreaks in the community at large are probably small compared to the indirect cost of other hospital-acquired infections that can require postdischarge treatment. However, these results demonstrate the direct effect of nosocomial gastroenteritis outbreaks on healthcare.

"Cost per bed-day" is a broad measure derived from the total net revenue expenditure divided by the total number of inpatient days (*26*). Thus, cost per bed-day includes overhead expenses, medical and nursing staff payroll, and equipment and treatment costs. U.K. guidelines stipulate that staff members or patients (ill or well) from affected units are to be excluded from unaffected units (*10*). In other words, as a measure to control further spread, reallocation of resources is prohibited by the guidelines. This restriction can be seen in terms of opportunity cost or a reduction from maximum efficiency. For example, although staff members who are not ill remain at work, they cannot be assigned to other units. Thus, when beds become empty, new admissions are restricted, and nursing services will not be used at maximum efficiency. The quantity of care will decrease, and the resources allocated towards such care will not decline.

Hospital-acquired infections have been estimated to cost the NHS £930 million (U.S. $1,488 million) annually (*32*). If these costs are distributed evenly across the NHS, hospital-acquired infections would cost the three NHS Trusts (1.7% of all beds in the United Kingdom) in this study £16 million (U.S.$ 25.6 million). Our study suggests that gastroenteritis outbreaks account for 12.5% of that cost, and similar to urinary tract infections, are the most costly healthcare-acquired infection to NHS (32), costing £115 million (U.S.$ 184 million) or »1% of the total inpatient services budget. Attributing costs to healthcare-acquired infections is complex, particularly in the case of gastroenteritis outbreaks, because little of the added expense will go directly toward the affected patient.

Not admitting susceptible patients is an effective means of containing nosocomial norovirus outbreaks. Recognizing outbreaks of viral gastroenteritis in hospitals can be difficult because of the high frequency of incontinence and other causes of gastroenteritis, such as antimicrobial-associated diarrhea. ELISA diagnostic kits facilitate rapid diagnosis of norovirus infections (*17*). Hospital infection control teams should be encouraged to take fecal samples from patients with suspected cases of viral gastroenteritis and to seek diagnoses. A positive confirmation of norovirus should result in immediate restriction of new admissions to the affected unit. Our data suggest that this restriction can be achieved within 3 days of diagnosis of the first case, approximately 1 week of the outbreak's duration can be prevented.

High levels of bed occupancy, the large size of care units, and lack of isolation units in NHS hospitals may make them particularly vulnerable to norovirus outbreaks. Cohorting affected patients is difficult in English hospitals. Units are large (median 20 beds, in this study) and occupancy is very high (>95%), so patients are not easily maneuvered. Policies, procedures, and building design may have major effects on transmission of these infections and should be explored by epidemic modeling, institutional clinical trials, and international studies that analyze the effect of the environment. Thus, our cost estimates are specific to the context of the English NHS. A hospital system that operated at a lower bed-occupancy level or those with smaller numbers of beds per unit may be able to provide nursing care to affected patients, with minimal effect on occupancy or other hospital processes. Economic analyses should be specifically tailored to the healthcare system that is being assessed.

Previous studies on the cost of hospital-acquired infections may have underestimated the effect of gastrointestinal infections because surveillance methods for such outbreaks have historically been lacking. This analysis of an active, enhanced surveillance scheme of three major hospital administrations in a defined geographic area quantifies the cost of gastroenteritis outbreaks to the health service, the important etiologic role of noroviruses, and the positive effect of control efforts.
